# A 32-Bit Single Quadrant Angle-Controlled Chipless Tag for Radio Frequency Identification Applications

**DOI:** 10.3390/s22072492

**Published:** 2022-03-24

**Authors:** Muhammad Noman, Usman A. Haider, Hidayat Ullah, Farooq A. Tahir, Hatem Rmili, Ali I. Najam

**Affiliations:** 1 Research Institute for Microwave and Millimeter-Wave Studies, School of Electrical Engineering and Computer Science, National University of Sciences and Technology, Islamabad 44000, Pakistan; munoman.msee18seecs@seecs.edu.pk (M.N.); uhaider.msee18seecs@seecs.edu.pk (U.A.H.); hidayat.ullah@seecs.edu.pk (H.U.); 2 K.A. CARE Energy Research and Innovation Center, King Abdulaziz University, Jeddah 21589, Saudi Arabia; hmrmili@kau.edu.sa; 3 Electrical and Computer Engineering Department, Faculty of Engineering, King Abdulaziz University, Jeddah 21589, Saudi Arabia; 4 National Electronics Complex of Pakistan, Islamabad 44000, Pakistan; alimranajam@gmail.com

**Keywords:** chipless RFID, product identification, miniaturized, barcode, future systems, IoT

## Abstract

A 32-bit chipless RFID tag operating in the 4.5–10.9 GHz band is presented in this paper. The tag has a unique multiple-arc-type shape consisting of closely packed 0.2 mm wide arcs of different radii and lengths. The specific tag geometry provides multiple resonances in frequency domain of an RCS plot. A frequency domain coding technique has also been proposed to encode the tag’s RCS signature into a 32-bit digital identification code. The tag has an overall dimension of 17.9 × 17.9 mm^2^, resulting in a high code density of 9.98 bits/cm^2^ and spectral efficiency of 5 bits/GHz. The proposed tag is built on a low loss substrate bearing a very small footprint, thereby making it extremely suitable for large-scale product identification purposes in future chipless RFID tag systems.

## 1. Introduction

Optical barcode technology has been used for decades now for the identification, monitoring, reading, and tracking of items in various scenarios [1]. The barcode technology is low cost and reliable; however, the latest developments in communications, computing, and automated systems have raised concerns about the limitations of optical barcode systems. These limitations include primarily the inevitable line of sight (LoS) and strict human–machine interface (HMI) requirements. Furthermore, barcode technology has low security and is short ranged. These limitations can be overcome by using wireless radio frequency identification (RFID) [2]. An RFID system uses a transceiver system and an electronic tag to identify a target item. Applications of RFID include shopping products, cargo items, employee’s card reading, vehicle identification, etc. In the near future, it is expected that billions of various different products will use RFID tags [3].

An RFID system provides higher security, longer range, high data capacity, and automated operation as compared to the existing optical barcode systems. The tag used in RFID system are of two types: (1) with on-board electronics and (2) without any electronics. The latter being called a chipless RFID tag and is preferred over the former due to its extremely low cost. The chipless RFID is, therefore, under great focus in research community so that it can be enabled to be used in future product identification systems. Since a chipless RFID (CRFID) tag does not use any onboard electronics, the data encoding becomes quite a challenge. Various encoding schemes have been proposed for CRFID systems, out of which four techniques are very prominent [4]. These are: (1) the Time-domain technique, (2) the Frequency-domain technique, (3) the Spatial-domain technique, and (4) the Hybrid technique [5,6,7,8,9,10,11,12,13,14]. The frequency domain technique works on backscattering phenomena in which a transmitted wave reflects from a metallic tag and its radar cross-section (RCS) is computed. The RCS contains encoded information about the tag in question. Several CRFID tags based on these frequency domain (FD) techniques have been proposed [15,16,17,18,19,20,21,22,23,24,25,26,27,28,29,30,31,32,33,34,35] to cope with challenges such as: code density, spectral efficiency, conformability, and cost.

Recently published FD-based CRFID tags are mostly based on closed loop resonators, such as [23], which is an elliptically shaped tag having a code density of 2.74 bits/cm^2^ and a spectral efficiency of 0.83 bits/GHz operating in 3.5–15.5 GHz band. A trefoil-shaped tag of [24] operating in 5.4–10.4 GHz band bears an overall size of 13.55 × 13.55 mm^2^. This tag has a code density of 5.44 bits/cm^2^ and spectral efficiency of 2 bits/GHz. Similarly, a butterfly [25] and kite-shaped [26] tags with high bit densities of 5.1 and 5.44 bits/cm^2^ respectively were presented. Both tags operate in 4.7–10 GHz and attain a low-spectral efficiency of 2 bits/GHz. These tags [23,24,25,26] have high bit densities due to their compact sizes and complex structures; however, they compromise their bit spectral efficiencies on the other hand.

Open-loop-resonator-based CRFID tags [27,28,29,30,31,32,33,34,35] have also been reported recently. For example, an 8-bit L shaped tag presented in [27] has a code density of 4 bits/cm^2^ and spectral efficiency of 5.33 bits/GHz. Semi-Elliptical Shaped tag in [28] provides a code density of 4.7 bits/cm^2^ and low spectral efficiency of 1.68 bits/GHz. A dipole-based tags with capacitive loading [29] provides high spectral efficiency of 12.5 bits/GHz, but it has very low code density of 1.77 bits/cm^2^. This is because of its very large size of 16.7 × 67.8 mm^2^. An 8-bit orientation independent circular ring slot-based tag is presented in [30]. The tag has a large area of 60.84 mm^2^ operating in 6–13 GHz that results in very low code density and spectral efficiency of 3.26 bits/cm^2^ and 1.14 bits/GHz, respectively. Similarly, large size tags of [31,32] also results in very low bit densities. For example, coupled-line micro-strip-resonator-based tag [31] has a size of 60.3 × 11 mm^2^ and a code density of 1.1 bits/cm^2^. A rectangular ring slot-based resonator [32] has a simple design with a size of 35 × 35 mm^2^, providing a very low 0.98 bits/cm^2^ code density and a 1.9 bits/GHz of spectral efficiency.

In this paper, a miniaturized 17.9 × 17.9 mm^2^ novel 32-bit chipless RFID tag on an ultra-thin 0.127 mm substrate is presented. The tag geometry is designed such that the length of the resonators is angle-controlled in a single quadrant, rendering the tag an efficient design in respect with radar cross-section (RCS) responses with multiple angle-controlled resonances. Furthermore, the desired frequency spectrum is intelligently utilized resulting in high spectral efficiency. The novelty in the proposed design is two folds: (1) an angle-based geometry to intelligently produce a desired tag ID and (2) a customized encoding technique with unique categorization of frequency-RCS sample space.

## 2. Tag Design

### 2.1. Theoretical Tag Design and Coding Scheme

**Figure 1:**
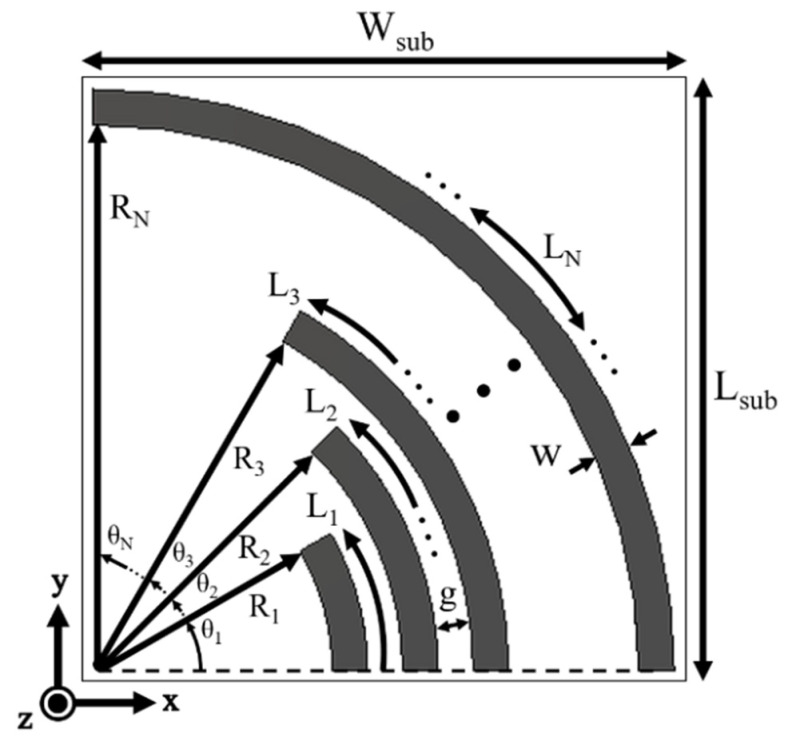
Proposed theoretical tag design.

The proposed tag is built upon a thin 0.127 mm Roger’s RT Duroid 5880 substrate with ε_r_ = 2.2 and tanδ = 0.0009. The theoretical geometry of the tag is shown in Figure 1, with an overall dimension of L_sub_ × W_sub_ mm^2^.

The top side of tag consists of ‘N’ number of arcs of arbitrary lengths ‘L_N_’. All these arcs exist in the first quadrant of a 2-dimensional plane having a radius R_N_. Each arc length starts at 0° from *x*-axis and terminate at an angle θ_N_. All arcs have a width of ‘w’ are separated by a distance ‘g’. The back side of proposed tag does not contain any copper layer and remains empty. The idea behind using multiple arcs of various lengths is that each arc will produce an RCS resonance in the frequency domain, thereby enabling the realization of a single logical bit.

**Figure 2:**
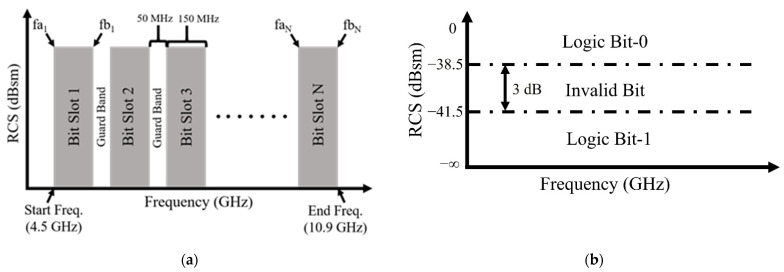
(**a**) Spectral allocation for the proposed tag; (**b**) RCS magnitude allocation for the proposed tag.

The coding scheme used for the proposed tag is frequency domain coding, for which the spectral allocation is shown in Figure 2a. The operating band chosen for this particular tag design is 4.5 to 10.9 GHz. A frequency slot of 150 MHz has been designated to indicate the existence of a valid logical bit, known as a ‘bit slot’. Hence, there are a total of ‘N’ bit slots within the operating band. Each ‘bit slot’ is separated by a ‘guard band’ of 50 MHz. The guard bands will be used to separate the identification of a true and a false bit-reading. The most significant bit is chosen to be ‘bit slot 1’, whereas ‘bit slot N’ is the least significant bit in an N-bit ID code. A bit slot start frequency is indicated by ‘fa’ and its stop frequency by ‘fb’ with a subscript ‘N’ showing its bit-position.

To further enhance bit-reading mechanism, the vertical RCS axis has been divided into three main regions: (1) a valid logic bit-0 region, (2) a valid logic bit-1 region, and (3) an invalid bit region. This division of RCS magnitude is shown in Figure 2b. The first designated RCS magnitude region (i.e., for logic bit-0) is from 0 to −38.5 dBsm. The second designated RCS magnitude region (i.e., for logic bit-1) is from −41.5 dBsm to −infinity, whereas the middle region of 3-dB separation from −38.5 to −41.5 dBsm has been designated for an invalid bit reading, i.e., a false logic bit.

The proposed tag is being designed to store a 32-bit digital ID code, therefore, variable N = 32. To realize a 32-bit code of any value, an RCS resonance or an RCS maximum must be produced at designated regions introduced in Figure 2a,b. For example, if a logical bit-1 is desired at any bit slot, an RCS resonance will have to be produced at the central frequency of that particular bit slot. A central frequency is located at +75 MHz from the start frequency of any bit slot. Similarly, if a logical bit-0 is desired at any bit-slot, an RCS maximum will have to be produced at the central frequency of that particular bit slot. Furthermore, an RCS maximum or minimum (i.e., an RCS resonance) must also have a magnitude that falls within the designated RCS region identified in Figure 2b. The above-outlined bit-identification process can be summarized as laid out in Table 1.

To create a particular tag ID, the central frequencies ‘fc’ of each bit slot is noted against the required 32-bit number. The length of each arc is calculated using the following formula [36]:

(1) $$L_{N}=\frac{c}{2f}\sqrt{\frac{2}{\varepsilon_{r}+1}}$$

Once length of each arc has been calculated, its copper footprint is laid upon the substrate by using the following standard arc length formula:

(2) $$L_{N}=R_{N}\cdot \theta_{N}$$

where *R_N_* is the radius of arc with length *L_N_*, and *θ_N_* is its finishing angle in radians. It should be noted here that the starting angle for all the arc lengths is 0°.

### 2.2. Single-Bit Resonator

**Figure 3:**
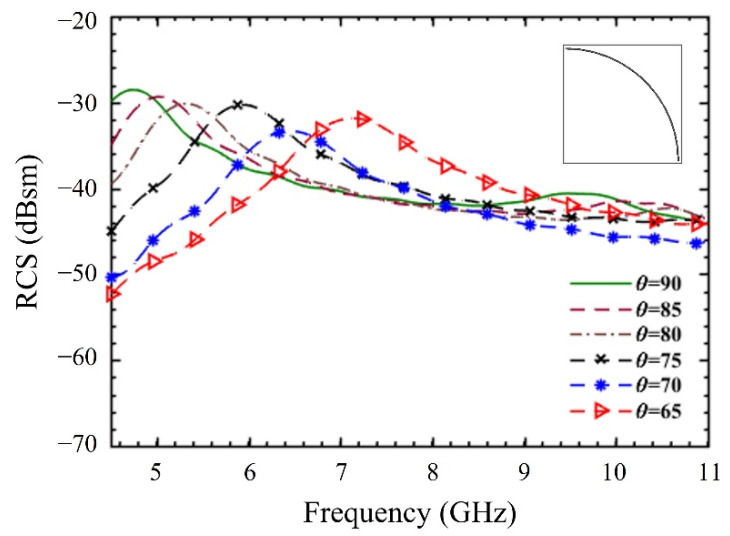
Effect of arc length over a single bit resonator.

To verify the effect of arc length over RCS, a single element tag is simulated. The parametric analysis of a single bit resonator is shown in Figure 3, in which the inset shows the geometric shape of the arc on a tag. It should be noted here that according to Equation (2), the arc length is directly proportional to the arc’s subtended angle. Therefore, the RCS is plotted for various arc angles in Figure 3.

It can be clearly observed that as the length of the arc is varied (by changing θ), the RCS maximum shifts along the frequency axis. Consequently, it can be concluded that the RCS maximum is controlled by variations in length L (or angle θ) of the arc. Contrary to this, an absence of an arc element will result in an RCS minimum. The RCS minimum in this manuscript will be called an “RCS resonance”. In the proposed design, the width ‘w’ and gap ‘g’ between the arc elements have been chosen to be 0.2 mm.

The RCS for single arc length copper strip depicted in Figure 3 is spread out wide around its maxima. This indicates low Q for the resonator, which has an advantage of high RCS magnitude. An excellent and detailed discussion about the relationship between RCS, resonance frequency, and quality factor using Characteristic Mode Theory (CMT) can be found in [37].

### 2.3. Tag Instances

Multiple tag instances of the theoretical design shown in Figure 1 have been simulated to show that any 32-bit ID can be realized. Keeping brevity, a list of only three tag names along with their 32-bit ID’s is shown in Table 2.

The first tag in the series, i.e., Tag 1, has been designed with an overall dimension of 17.9 × 17.9 mm^2^. The track width w = 0.2 mm, and the first element has been placed at a position where its radius *R*_1_ = 7.2 mm. The subsequent arc elements are placed at a distance *g* = 0.2 mm apart. The rest of the radii can therefore be calculated as follows:

(3) $$R_{N}=g+R_{N-1}$$

where *R_N_* ≠ 1.

**Figure 4:**
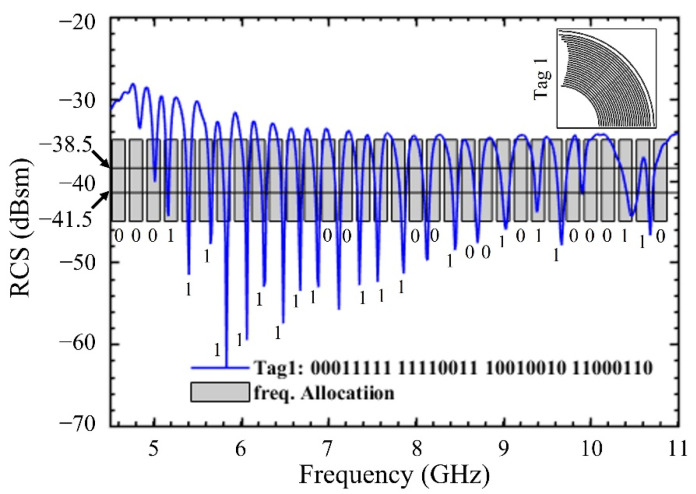
Simulated RCS response of Tag 1.

It has been found through simulation that a total of 24 arc elements were sufficient to generate the 32-bit ID mentioned in Table 2, against Tag 1. An extra arc at a distance g = 0.3 mm has been placed to balance the coupling effect between the rest of the arc elements. The resulting tag geometry and its RCS response is shown in Figure 4.

The bit values against each bit slot have been properly labeled according to the criterion set in Table 1. The most significant is at the lowest frequency of 4.55 GHz, whereas the least significant bit occurs at the highest frequency of 10.8 GHz. Frequency allocation as well as the RCS threshold regions have been shown in Figure 4 with a grayed rectangle and black dashed lines. A complete list of optimized arc lengths L1–L25 is given in Table 3.

**Figure 5:**
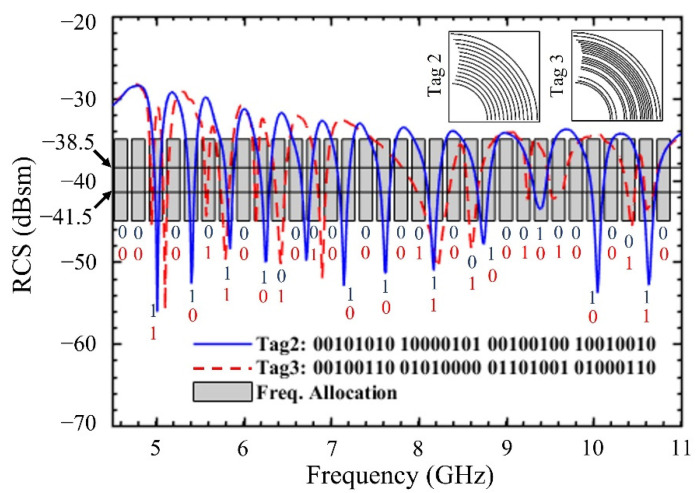
Simulated RCS response of CRFID tag instances: Tag 2, Tag 3. (Inset shows corresponding tag geometry).

The code density of the proposed tag is 9.98 bits/cm^2^ and a spectral efficiency of 5 bits/GHz. These values will remain valid for all the rest of tag instances. The remaining two tag instances outlined in Table 2 (i.e., Tag 2 and Tag 3) have also been simulated and are shown in Figure 5 along with their RCS responses. All these tags encode 32-bit IDs, however the number of elements used in each tag is different. For example, Tag 2 has 13 and Tag 3 has 18 arc elements, respectively. The dimensions of these tags remain the same, and hence their code densities, as well as spectral efficiencies, are unchanged to that of Tag 1. Some of state-of-the-artwork has been compared with this work and highlighted in Table 4.

## 3. Fabrication and Measurement Results

**Figure 6:**
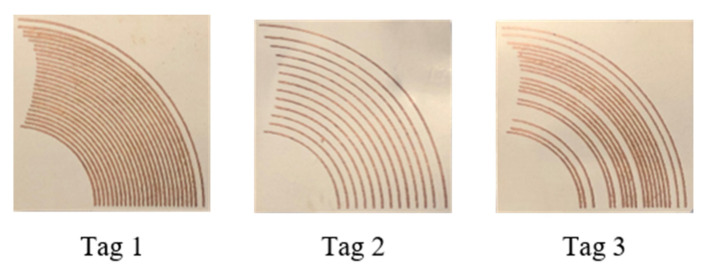
Photograph of fabricated CRFID tag instances.

**Figure 7:**
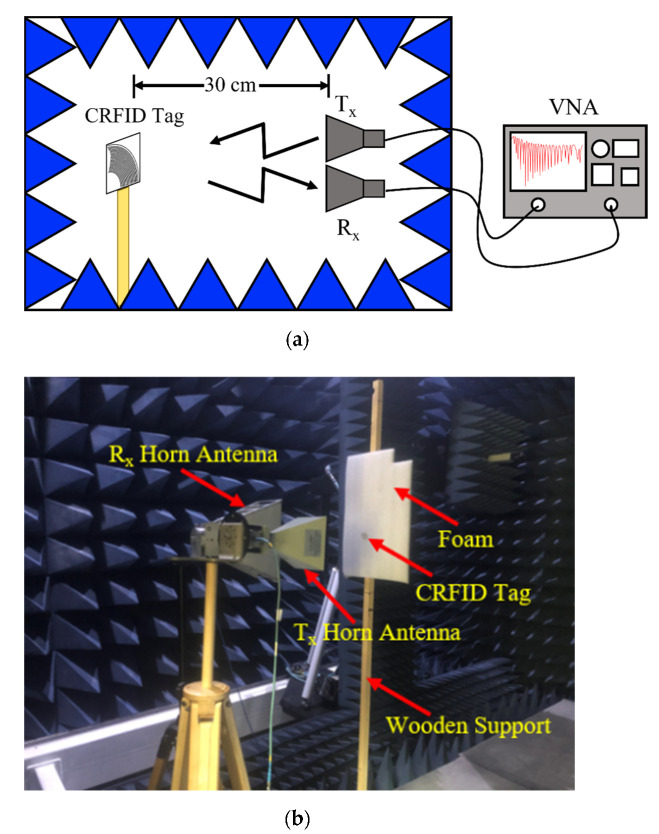
Measurement setup: (**a**) Schematic diagram; (**b**) Photograph of the measurement setup.

All three tags have been fabricated on an ultra-thin 0.127 mm Roger’s substrate, using standard PCB process. All tag instances were measured for RCS response in an anechoic chamber to verify the simulated response of the tag. The photographs of the fabricated tags are shown in Figure 6. A schematic of the measurement topology is shown in Figure 7a. The actual measurement setup in bi-static configuration in an anechoic chamber is shown in Figure 7.

The RCS of fabricated tags was measured using a standard formula provided by [38], which is as follows:

(4) $$\sigma^{\mathrm{tag}}={\left[\frac{S_{21}^{\mathrm{tag}}-S_{21}^{\mathrm{isolation}}}{S_{21}^{\mathrm{ref}}-S_{21}^{\mathrm{isolation}}}\right]}^{2}\cdot \sigma^{\mathrm{ref}}$$

where $S_{21}^{\mathrm{tag}}$ is measured S21 of the proposed tags, $S_{21}^{\mathrm{ref}}$ is the measured S-parameter of the reference rectangular metallic plate, $S_{21}^{\mathrm{isolation}}$ is the isolation measurement without the tag, $\sigma^{\mathrm{ref}}$ is the known RCS value of the reference rectangular metallic plate, and $\sigma^{\mathrm{tag}}$ is the obtained RCS value of the proposed chipless RFID tag.

**Figure 8:**
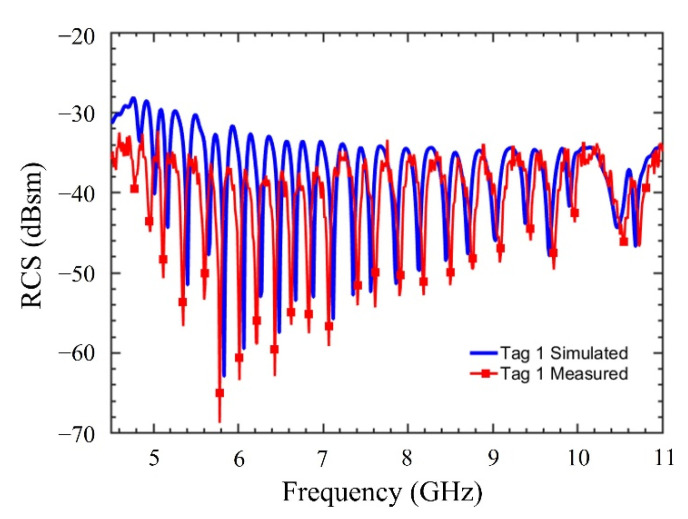
Measured and simulated RCS response of tag instance Tag 1.

**Figure 9:**
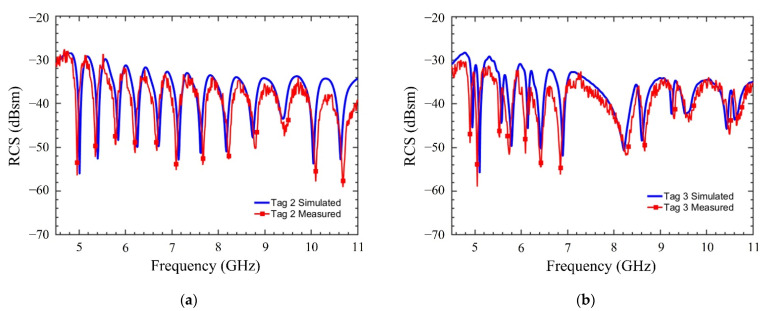
Measured and simulated RCS response of CRFID tag instances: (**a**) Tag 2; (**b**) Tag 3.

The experimentation to measure RCS response of the tags is performed using three different linearly polarized standard gain antennas (SGAs): (1) 3.95–5.85 GHz, (2) 5.85–8.20 GHz, and (3) 8.20–12.4 GHz. The SGAs are used in transmitting (Tx) mode and a broadband horn antenna (from 2–18 GHz) is used in the receiving (Rx) mode. The distance between the Tx and Rx antennas is kept around 0.75 m, which falls in the far-field region. The wideband horn and SGAs are connected to an Anritsu vector network analyzer (VNA) MS46122B. The SGAs have a gain of 12 dBi and the power delivered by VNA is 3 dBm. The simulated and measured RCS response of the Tag 1 is shown in Figure 8. Similarly, simulated, and measured RCS response of Tag 2–3 are shown in Figure 9. The measured results show a good correlation with those simulated.

## 4. Conclusions

A unique chipless RFID tag was introduced and analyzed in this paper. The tag consists of curved metallic resonating elements of various lengths that provide resonant points on RCS plot. This results in a tag which can encode a 32-bit digital number, operating in 4.5–10.9 GHz band. A special frequency domain coding method was also proposed that can potentially lead to an efficient decoding of the information stored in tag’s EM signature. The simulated and measured results show a stable RCS response with a code density of 9.98 bits/cm^2^ and spectral efficiency of 5 bits/GHz. The tag was built on an ultra-thin 0.127 mm Roger’s substrate. This unique tag is deemed very suitable for future product identification and IoT systems designed with chipless RFID tags capabilities.

## Figures and Tables

**Table 1:** Summary of bit decoding process.

Case	Frequency Range, f (GHz)	RCS Magnitude Range (dBsm)	Decoded Logic
1	Fa_N_ < f < Fb_N_	|RCS| > −38.5	0
2	Fa_N_ < f < Fb_N_	|RCS| < −41.5	1
3	Fa_N_ < f < Fb_N_	−38.5 > RCS > −41.5	invalid
4	Fa_N−1_ < f < Fa_N_	RCS > 0	invalid

**Table 2:** List of tags and their IDs.

Sr. No.	Tag Name	Tag 32-Bit ID
1	Tag 1	00011111111100111001001011000110
2	Tag 2	00101010100001010010010010010010
3	Tag 3	00100110010100000110100101000110

**Table 3:** List of optimized parameters for Tag 1.

Parameter	L1	L2	L3	L4	L5
Value (mm)	11.22	11.45	11.98	12.5	13.03
Parameter	L6	L7	L8	L9	L10
Value (mm)	13.46	13.98	14.56	15.14	15.72
Parameter	L11	L12	L13	L14	L15
Value (mm)	16.3	16.82	17.4	18.01	18.6
Parameter	L16	L17	L18	L19	L20
Value (mm)	19.21	19.97	20.6	21.43	22.05
Parameter	L21	L22	L23	L24	L25
Value (mm)	23.18	24.31	24.94	26.07	27.17

**Table 4:** Comparison with state-of-the-art CRFID tags.

Ref. No.	Operating Frequency (GHz)	No. of Bits	Code Density (Bits/cm^2^)	Spectral Efficiency (Bits/GHz)
[29]	2–3.6	20	1.77	12.5
[30]	6–13	8	3.26	1.14
[33]	2.2–3.5	20	0.6	15.4
[34]	4.5–7.5	14	5.88	4.66
[35]	2–8	8	10.74	1.33
Proposed	4.5–10.9	32	9.98	5

## Data Availability

Not applicable.
